# *PHOX2B* defects alter protein folding, cell-cycle, and mitochondrial pathways in an in vitro model of CCHS

**DOI:** 10.1186/s10020-026-01498-1

**Published:** 2026-05-18

**Authors:** Chiara Africano, Tiziana Bachetti, Eleonora Di Zanni, Giuseppe Santamaria, Roberto Cusano, Ignazia Prigione, Genny Del Zotto, Paolo Uva, Isabella Ceccherini

**Affiliations:** 1 UOSD Aggregation area of Research Laboratories, IRCCS Istituto Giannina Gaslini, Genova, 16147 Italy; 2 IRCCS Azienda Ospedaliera Metropolitana, Genova, 16132 Italy; 3 Department of Anesthesiology, Weill Cornell Medical College, New York, NY USA; 4 Centro di Ricerca, Studi Superiori in Sardegna (CRS4), Sviluppo, Pula, Cagliari, 09100 Italy; 5 UOC Rheumatology and Autoinflammatory Diseases, IRCCS Istituto Giannina Gaslini, Genova, 16147 Italy; 6 Core Facilities, Department of Research and Diagnostics, IRCCS Istituto Giannina Gaslini, Genova, 16147 Italy; 7 Clinical Bioinformatics Unit, IRCCS Istituto Giannina Gaslini, Genova, 16147 Italy

**Keywords:** Congenital central hypoventilation syndrome, Transcriptome analysis, Altered pathways

## Abstract

**Background:**

Congenital Central Hypoventilation Syndrome (CCHS) is a rare autosomal dominant disorder caused by heterozygous mutations in the *PHOX2B* gene, leading to impaired ventilatory responses to hypoxia and hypercapnia. No pharmacological therapy exists, and patients rely on ventilatory support, tracheostomy, or diaphragmatic pacing. Most mutations are polyalanine expansions in exon 3, which mislocalize PHOX2B to the cytoplasm, disrupting the regulation of its transcriptional targets. Previous studies showed that geldanamycin and its derivative 17-AAG can partially rescue both the localization and the function of polyalanine expansion mutant PHOX2B. Nonetheless, downstream molecular effects of these mutations remain poorly understood.

**Methods:**

The transcriptomic approach was applied to cells transiently expressing wild-type PHOX2B or the mutant carrying the most severe polyalanine expansion, to investigate the cellular consequences of whole transcripts deregulation caused by the *PHOX2B* mutation with or without 17-AAG treatments.

**Results:**

Bioinformatic analysis allowed us to confirm the involvement of pathways already observed in polyalanine pathogenesis, such as protein folding and transcriptional repression, and to identify oxidative stress, mitochondrial dysfunction, and altered cell-cycle regulation as novel components of the PHOX2B+13Ala pathogenesis.

**Conclusions:**

The RNA-sequencing approach recapitulates the molecular pathogenesis of PHOX2B polyalanine expansion mutations in CCHS and in vitro functional validations, thus confirming the suitability of this cellular model to study the molecular pathogenesis of the disease.

**Supplementary Information:**

The online version contains supplementary material available at 10.1186/s10020-026-01498-1.

## Background

Congenital Central Hypoventilation Syndrome (CCHS, MIM 209880) is a rare congenital / neonatal autosomal dominant genetic disorder characterized by an abnormal ventilatory response to hypoxia and hypercapnia, mainly during sleep but in the most severe cases also during wakefulness. Unfortunately, no pharmacological strategy is available and patients are currently managed by ventilator, tracheostomy or diaphragmatic pacer supports (Di Lascio et al. 2021; Slattery et al. 2023; Trang et al. 2020).

CCHS is caused by heterozygous mutations of the *PHOX2B* gene (Amiel et al. 2003; Zhou et al. 2021). In frame triplet duplications in exon 3, leading to expansions of a 20 polyalanine (polyA) tract from + 4 to + 13 additional residues, also defined as PolyAlanine Repeat Mutations (PARMs), account for about 90% causative *PHOX2B* mutations. In addition, rare missense, nonsense, and frameshift *PHOX2B* mutations can also occur in CCHS patients (Bachetti and Ceccherini 2020).

In vitro studies have shown that the respiratory phenotype relies on the aberrant retention of the PHOX2B protein in the cytoplasmic compartment, in diffuse or aggregate forms depending on the extension of the polyA tract (Bachetti et al. 2005a). This effect results in impaired transactivation of the *PHOX2B* gene itself and of the target genes identified so far such as *DBH*, *RET*, *TLX2*, *PHOX2A* (Bachetti et al. 2005b; Borghini et al. 2006; Di Lascio et al. 2013; Di Zanni et al. 2017). Furthermore, several studies have highlighted interactions between the polyalanine-expanded PHOX2B protein and the WT protein that underlie likely dominant-negative effects relevant to CCHS pathology (Di Lascio et al. 2016; Anton et al., 2024; Diana et al. 2024).

Of note, it has been shown that the stimulation of the cellular stress response, namely heat shock proteins, proteasome and autophagic pathways, by geldanamycin, 17-Allylaminogeldanamycin (17-AAG), and curcumin or HDAC inhibitor as SAHA, can prevent the formation of PHOX2B mutant aggregates, induces the clearance of intracellular inclusions by promoting the folding and/or elimination of mutant PHOX2B proteins, and rescues PHOX2B nuclear localization and transactivation of PHOX2B target genes by the mutant protein (Africano et al. 2024; Bachetti et al. 2007; Di Zanni et al. 2012). The E3-ubiquitin ligase TRIM11 was also shown to be effective in the proteasome-mediated clearance of polyAla PHOX2B proteins, thus confirming the role of this mechanism in the cellular response to PHOX2B misfolding (Parodi et al. 2012).

However, the vast majority of the network functionally connected to PHOX2B is still unknown, both in physiological and pathological conditions (Di Lascio et al. 2021). Unbiased approaches may result useful to broaden knowledge of PHOX2B-mediated molecular pathogenesis, a crucial step towards the development of a treatment strategy.

Here, we compare the transcriptomic profiles of cells expressing wild-type (WT) PHOX2B with those of cells expressing the severe + 13Ala expansion mutation. We also assess the effects of 17-AAG treatment on these profiles, evaluating its potential to restore a WT–like phenotype. Notably, beyond the established effects on protein folding and transcriptional impairment, our in vitro validation has now identified the oxidative stress, mitochondrial dysfunction, and altered cell-cycle regulation as novel contributors to PHOX2B+13Ala pathogenesis, providing new insights into the molecular mechanisms underlying this severe CCHS causing mutation.

## Methods

Human neuroblastoma SK-N-BE cells, transiently transfected with expression constructs encoding WT and + 13Ala PHOX2B, have undergone transcriptomic analysis to find genes differentially expressed between the two conditions and to perform the gene set enrichment analysis (GSEA). HeLa and SK-N-BE cell lines were used to experimentally validate the pathways involved in + 13Ala mediated pathogenesis, namely, ROS production and cell cycle progression, respectively. All cell lines were mycoplasma free.

All protocols and experimental details, including statistical analysis, are reported in the Supplementary Methods (Additional File 1).

## Results

### Experimental design of the study

To perform transcriptome analysis in cells expressing WT or + 13Ala PHOX2B proteins, the human neuroblastoma (NB) SK-N-BE cell line was selected for its low levels PHOX2B expression (Di Zanni et al. 2015), thus avoiding the detection of confounding effects, such as pathways constitutively up-regulated in the presence of a highly expressed PHOX2B protein. Additionally, in this cell line *PHOX2B* gene is not mutated and is present in single copy. For these reasons, it has already been used to detect PHOX2B downstream effects (Bachetti et al. 2005a; Di Zanni et al. 2017).

We used transcription datasets already generated and reported from SK-N-BE cells transfected with constructs expressing WT (“WT”) or mutant (“+13Ala”) PHOX2B proteins (Africano et al. 2024). These conditions had been applied in triplicate both without any treatment (“DMSO”) and in the presence of 17-AAG (“17AAG”), diluted in DMSO, for 24 h. This transcriptional dataset (hereinafter referred to as Set #1) was used to assess Gene Expression Profiles (GEPs) and to perform Gene Set Enrichment Analysis (GSEA). To validate the results thus obtained, two additional non replicated transcriptomic datasets, similarly obtained from SK-N-BE cells transfected with the empty vector, WT and + 13Ala constructs, and including the untransfected native cell line (hereinafter referred to as Sets #2 and #3 or “validation sets”), were used.

### Gene Expression Profiles (GEPs) induced by + 13Ala *PHOX2B* and regulated by 17-AAG treatment

Total RNA from all transfected/untransfected and treated/untreated cell lines was subjected to high-throughput RNA sequencing, generating ~ 30 million 75-bp single-end reads per sample. After quality control, reads were aligned to Ensembl transcripts (GRCh38) and summarized at the gene level, assessing ~ 36,000 genes (Love et al. 2014; Wang et al. 2012). This dataset (Set #1) had already been used to identify differentially expressed genes (DEGs) and key pathways during a previous study via Cytoscape-based network analysis (Africano et al. 2024; Bindea et al. 2009; Kohl et al. 2011).

**Fig. 1:**
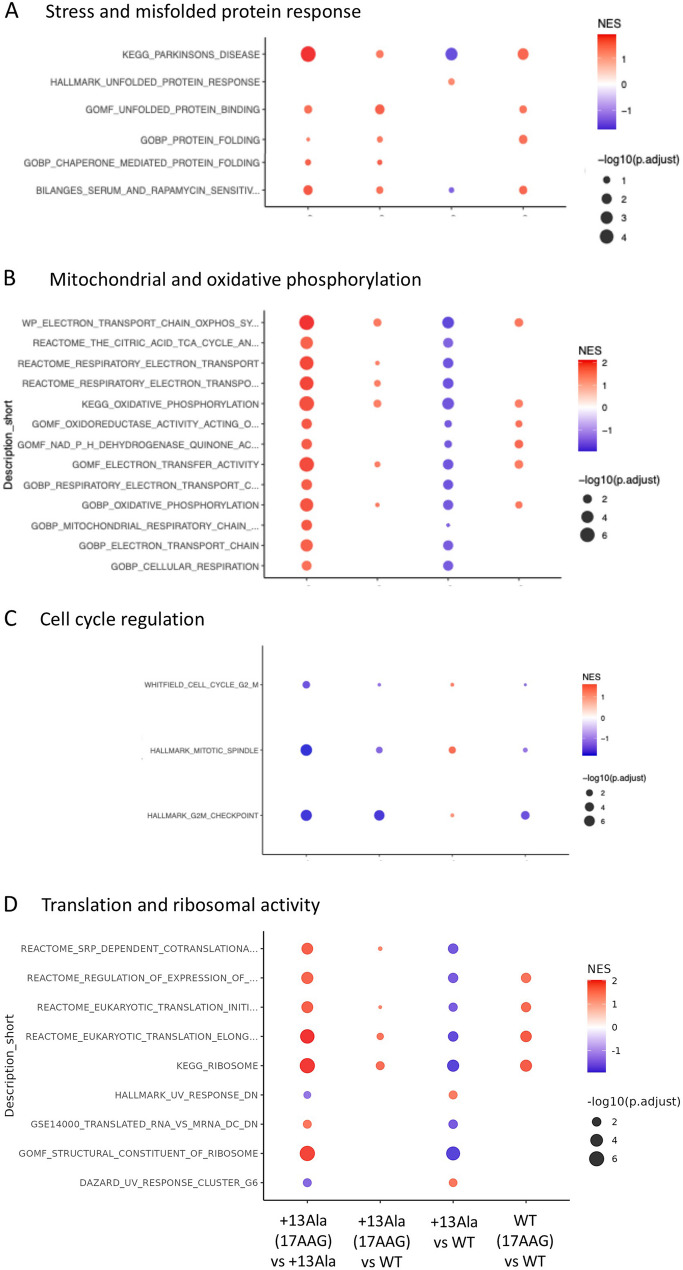
GSEA analysis. Representation of four sets of pathways (A-D) dysregulated by PHOX2B+13Ala and reverted upon 17-AAG treatment. **A** Mechanisms of misfolded protein response. **B** Cellular respiratory reactions. **C** Cell cycle. **D** translation and gene expression regulations. Pathways were analysed in 4 comparisons (X-axis, from left to right): +13Ala(17AAG) vs. + 13Ala, + 13Ala(17AAG) vs. WT, + 13Ala vs. WT, and WT(17AAG) vs. WT. Those reported are significant for at least one comparisons between different conditions, using p.adjust < 0.05 as treshold. Dots in red and blue indicate pathways that are up-regulated and down-regulation, respectively. The size of the dot corresponds to–log10(p.adjust) value

In an attempt to uncover additional molecular mechanisms influenced by the + 13Ala mutation and ultimately modulated by 17-AAG, a gene set enrichment analysis (GSEA) was performed during the current study using four MSigDB collections: Hallmark (H), Chemical and Genetic Perturbations (CGP), GO Biological Process (GO), and Canonical Pathways (CP) [1]. We focused on pathways that mediate the most deleterious effects of PHOX2B +13Ala and are potentially reversible by 17-AAG. For each collection, top up- and down-regulated signatures were ranked across the comparisons: +13Ala vs. WT, + 13Ala(17AAG) vs. WT, + 13Ala(17AAG) vs. + 13Ala, and WT(17AAG) vs. WT. Signatures with opposite effects between the mutation and the treatment were graphically represented (Fig. 1), with dot color indicating direction (red = up, blue = down; NES) and size corresponding to statistical significance (–log10 p.adjust).

In accordance with the previous Cytoscape-based network analysis (Africano et al. 2024), GSEA analysis allowed us to confirm the involvement of some pathways already known in the pathogenesis mediated by PHOX2B polyAla mutations in vitro. As expected, protein aggregation, misfolded or unfolded protein response, proteasome and autophagy process have confirmed to be reversed by 17-AAG treatment (Fig. 1A), as well as the downregulation of cellular respiration and oxidative stress, such as the electron transport chain, oxidative phosphorylation, and the citric acid cycle, observed in + 13Ala compared to WT (Fig. 1B).

The pathways related to cell cycle (particularly the G2-M checkpoint) and mitotic spindle are shown slightly up-regulated and reversed by 17-AAG (Fig. 1C). Finally, the same treatment has shown beneficial effects also on the + 13Ala mediated down-regulation in gene expression and translation process (Fig. 1D).

These results confirm the feasibility and suitability of the transcriptomic approach to unravel both new and already known pathogenic mechanisms playing a role in CCHS.

### Pathways differentially expressed by PolyA in two previous transcriptomics datasets

In an attempt to confirm the RNA-seq and GSEA results obtained from Set #1, in which clear activation of mitochondrial pathways, cell cycle programs, and stress response signatures were also observed, we examined two validation datasets (Sets #2 and #3), which included cells expressing PHOX2B WT and + 13Ala, along with untransfected control cells or cells transfected with an empty vector.

**Fig. 2:**
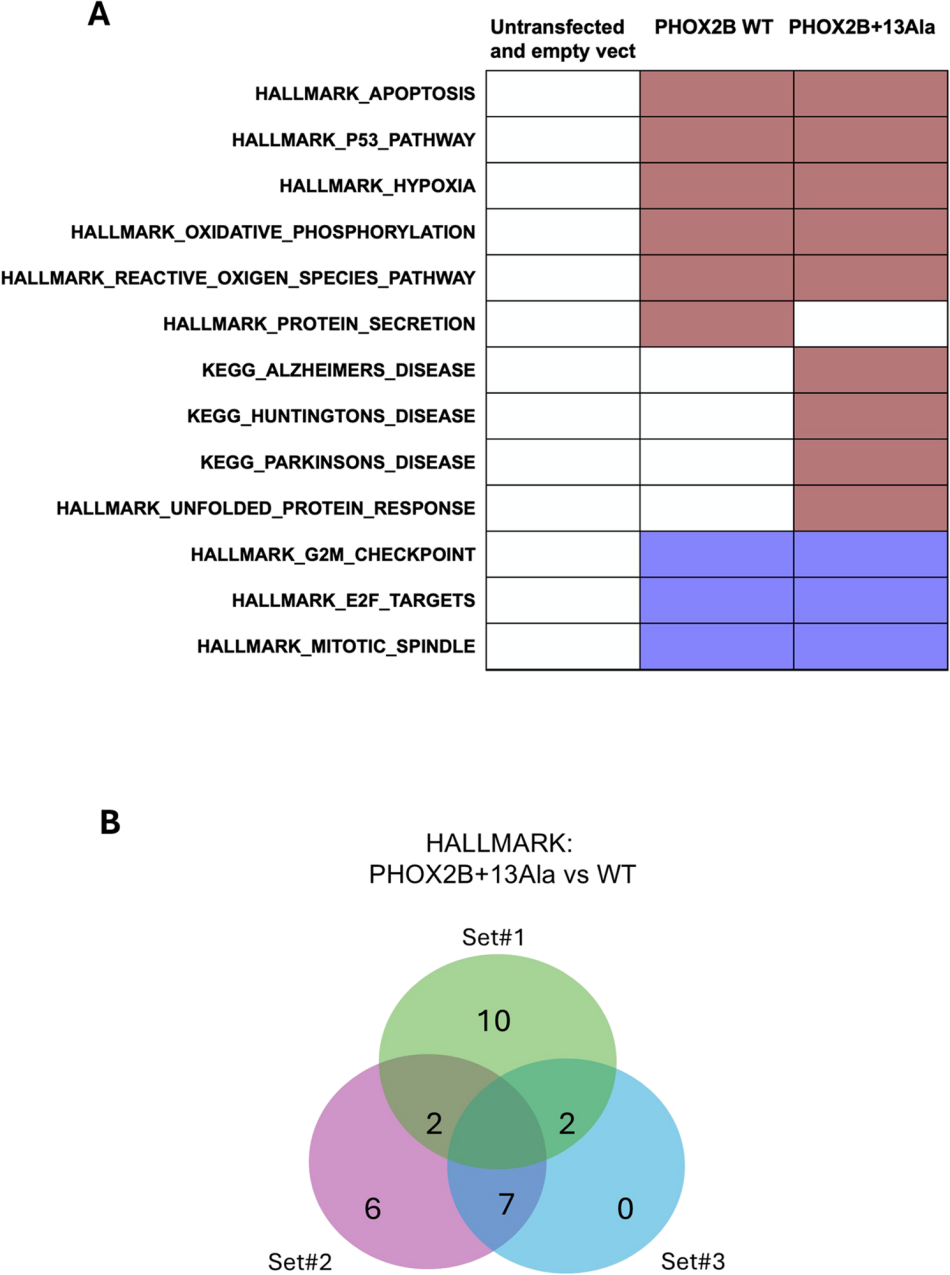
GSEA analysis of the validation datasets, comparing PHOX2B WT, + 13Ala and controls (untrasfected and transfected with an empty vector).** A** In the two heatmap the top ranked pathways are reported on the left and the conditions (controls, PHOX2B WT, and PHOX2B+13Ala) are shown on the top. The blue colour indicates the down-regulation of the genes related to the pathways, while the red colour represents the upregulated processes. Baseline levels are left blank. **B** Venn Diagram of “Hallmark” pathways specific and shared by Set#1, Set#2 and Set#3 comparing PHOX2B+13Ala and WT

We first compared these two latter control conditions (untransfected and transfected with empty vector) finding almost identical profiles characterized by a broad downregulation of the pathways previously found to be upregulated in the presence of PHOX2B + 13Ala. This indicates that the transfection procedure itself did not induce detectable metabolic, stress-related, or proliferative responses (Fig. 2A), providing a robust baseline for assessing the impact of PHOX2B expression.

In contrast, cells overexpressing PHOX2B WT exhibited an activation across multiple pathway categories, including oxidative responses, hypoxia-related signatures, unfolded protein response (UPR), proteostasis, and cell-cycle programs. Since none of these changes was observed in the control conditions, they must reflect a specific biological response to PHOX2B overexpression rather than a transfection artifact. At the same time, cell cycle pathways were found to be strongly downregulated (Fig. 2A). The PHOX2B + 13Ala variant triggered the same general response (Fig. 2A), but at a different level, as oxidative stress and UPR signatures were even more strongly upregulated than the WT variant and also compared to transcriptional hallmarks of the misfolded protein response and neurodegenerative and triplet-expansion diseases (Alzheimer’s, Huntington’s, and Parkinson’s).

To further explore such a differential expression, we performed a comparative GSEA analysis on cells expressing the WT and expanded PHOX2B proteins. As shown in Table 1, both Set #2 and Set #3 exhibited shared significant enrichment of pathways associated with mitochondrial activity and oxidative metabolism, such as oxidative phosphorylation, responses to hypoxia, and pathways involving reactive oxygen species. This finding, consistent across the three datasets, supports the idea that mitochondrial remodeling is a stable and reproducible feature of this CCHS cellular model.

**Table 1:** GSEA analysis of Set #2 and Set #3 (PHOX2B+13Ala vs WT)

*Biological*	*Pathway Example*	*Set #2 (PHOX2B*	*Set #3 (PHOX2B*	*Shared*
*Process*		*+13Ala vs WT)*	*+13Ala vs WT)*	
Mitochondrial activity	HALLMARK_HYPOXIA	up	up	shared
	HALLMARK_REACTIVE_OXIGEN_SPECIES_PATHWAY	up	up	shared
Cell cycle	HALLMARK_G2M_CHECKPOINT	--	up	specific to Set #3
	HALLMARK_MITOTIC_SPINDLE	--	up	specific to Set #3
	HALLMARK_E2F_TARGETS	up	up	shared
	KEGG_CELL_CYCLE	up	up	shared
	REACTOME_CELL_CYCLE	up	up	shared
	REACTOME_CELL_CYCLE_CHECKPOINTS	up	up	shared
	REACTOME_CELL_CYCLE_MITOTIC	up	up	shared
	REACTOME_REGULATION_OF_MITOTIC_CELL_CYCLE	up	up	shared
Genome maintenance	HALLMARK_P53_PATHWAY	up	up	shared
	HALLMARK_DNA_REPAIR	up	up	shared
	REACTOME_DNA_REPLICATION	up	up	shared
Cellular growth/ stress	HALLMARK_MYC_TARGETS_V1	up	up	shared
	HALLMARK_MYC_TARGETS_V2	up	up	shared
	HALLMARK_MTORC1_SIGNALING	up	up	shared
	HALLMARK_TNFA_SIGNALING_VIA_NFKB	up	--	specific to Set #2
Translation/protein metabolism	KEGG_RIBOSOME	up	up	shared
	REACTOME_METABOLISM_OF_PROTEINS	up	up	shared
	REACTOME_TRANSLATION	up	up	shared
Unfolded protein response	BIOCARTA_PROTEASOME_PATHWAY	up	up	shared
	KEGG_PROTEASOME	up	up	shared
	KEGG_ALZHEIMERS_DISEASE	up	up	shared
	KEGG_HUNTINGTONS_DISEASE	up	up	shared
	KEGGS_PARKINSONS_DISEASE	up	up	shared

Broad activation of cell-cycle and DNA-maintenance programs was also observed in both validation sets, in line with what detected in the previously reported RNA-seq dataset (Set #1). While most cell-cycle pathways were shared between the two analyses, Set #3 uniquely displayed enrichment of G2/M checkpoint and mitotic spindle signatures (Table 1).

Pathways linked to cellular growth and stress responses, such as MYC targets and mTORC1 signalling, were commonly enriched across the datasets, while TNFα/NF-κB signalling was present only in Set #2.

Finally, signatures associated with protein synthesis, proteostasis and the unfolded protein response, including ribosome- and proteasome-related pathways, syndromes caused by protein aggregates deposits such as Huntington or Parkinson’s diseases, were consistently detected in both sets #2 and #3. These patterns echo the stress-adaptive aspects observed in the previous RNA-seq/GSEA analysis of Set #1, pointing to robust activation of mechanisms regulating protein turnover and quality control.

Overall, the strong concordance between the two validation sets and the GSEA results from Set #1 provides compelling evidence of the reproducibility of these biological responses, strengthening the interpretation of the core pathways altered under polyA conditions. This evidence is shown in Fig. 2B with a representation of shared Hallmark pathways among the three datasets in the PHOX2B+13Ala vs. WT comparison.

Together, these results demonstrate that the pathway shifts observed are not incidental nor attributable to transfection toxicity, but instead reflect a genuine and reproducible biological effect specifically driven by PHOX2B, with the + 13Ala variant inducing a markedly enhanced response.

Some of the mechanisms highlighted by the analysis described above are known and already demonstrated, such as the effects due to misfolding induced in vitro by alanine expansions. Others awaited experimental confirmation, such as ROS production, while the involvement of the cell cycle had not yet been considered. Below, we report the results of an experimental investigation of these latter mechanisms, as they appear to contribute in vitro to the effects of overexpression of PHOX2B and its + 13Ala variant.

### Evaluation of ROS production

Reactive oxygen species (ROS) production is a regulated process occurring at all cellular stages under both physiological and pathological conditions. During hypoxia, the composition of electron transport chain (ETC) complexes is modified through subunit alterations, exchange, or depletion to limit excessive ROS generation and prevent cellular damage (Fuhrmann and Brüne 2017).

In our study, RNA-seq analysis revealed that the respiratory electron transport chain is down-regulated by + 13Ala and can be rescued by treatment with 17-AAG. It is known that dysfunction of ETC can increase the production of ROS leading to cellular oxidative stress and mitochondrial damage (Quoilin et al. 2014).

Our attention was attracted by hypoxia related perturbations since the ROS presence has already been detected in blood samples and urine of CCHS patients and PHOX2B+13Ala has been observed to induce misfolding and aggregation, i.e. processes that are often coupled with oxidative stress in cells (Abramov et al. 2020; Bigagli et al. 2022; Degl’Innocenti et al. 2018).

**Fig. 3:**
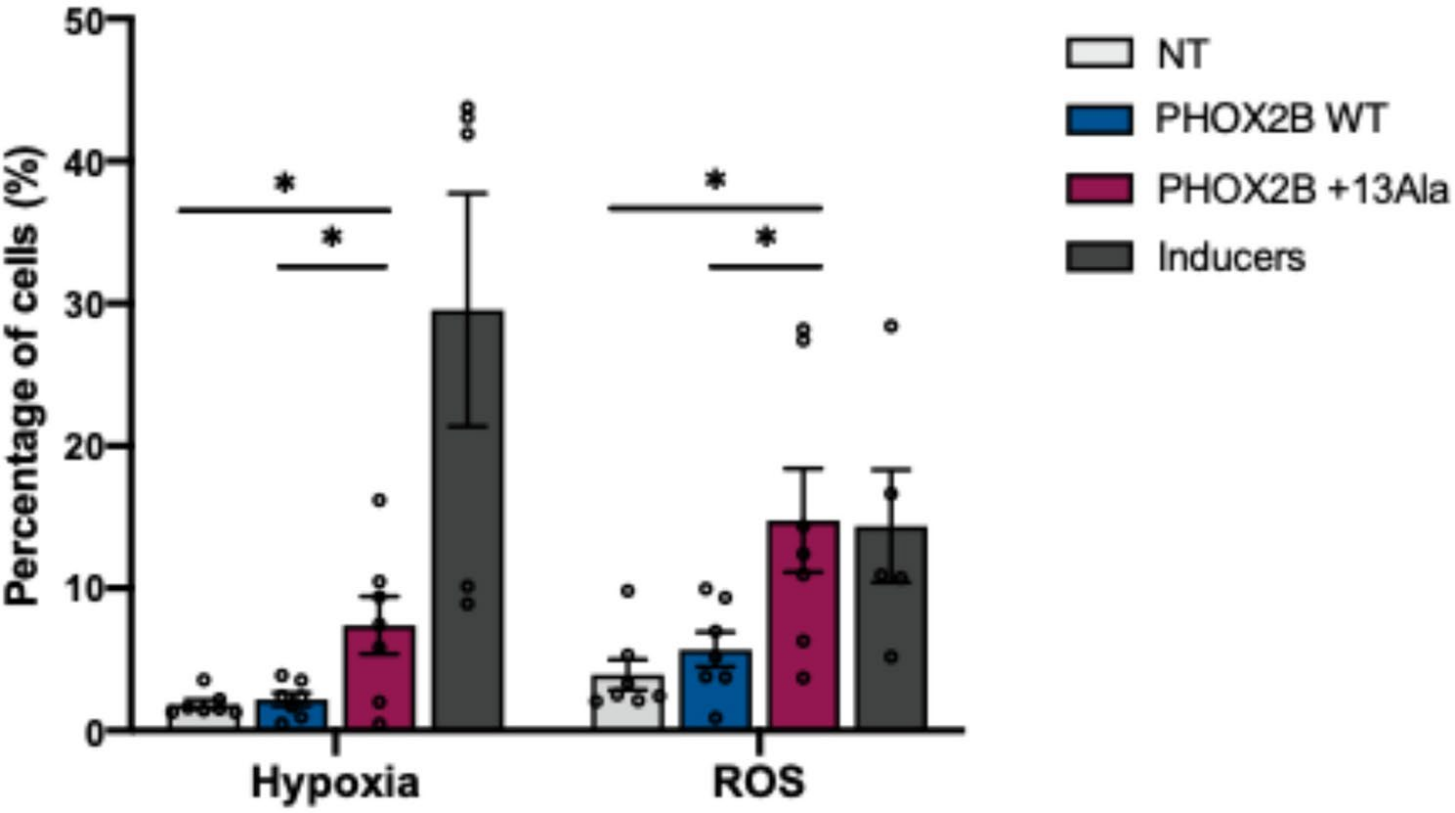
Evaluation of Hypoxia response and ROS production. Number of hypoxic or oxidative stress positive cells was measured cytometrically 48 h post-transfection. Histograms represent differences in percentages of cells carrying PHOX2B WT and + 13Ala, that produce ROS or are in a condition of hypoxic stress, compared to untransfected cells (NT = Not Transfected) and to inducers (DFO as hypoxia inducer, PYO as ROS inducer). Values are the mean ± SEM of *n* = 7 independent experiments. Statistical analysis was performed by two-way ANOVA with Sidak’s post hoc multiple comparisons test (*p* < 0.05)

To confirm and validate the involvement of the stress response in PHOX2B expressing cells (WT and + 13Ala), we transfected Hela cells with constructs pcDNA3.1Myc-*PHOX2B WT* and pcDNA3.1Myc-*PHOX2B+13Ala*. The hypoxic and oxidative stress response analysis, carried out cytometrically 48 h post-transfection, revealed that polyalanine expanded PHOX2B increased both levels of intracellular ROS production and hypoxic stress-induced cells compared to the wildtype condition (Fig. 3) (*p* < 0.05). In particular, ROS levels in + 13Ala are comparable to ROS produced in the positive control, i.e. cells treated with a specific ROS inducer (Pyocyanin-PYO).

These findings suggest a shift from an oxidative, respiration-competent metabolic state (PHOX2B WT) toward a pseudo-hypoxic condition in PHOX2B+13Ala-expressing cells, thus confirming previous reports in CCHS patients (Bigagli et al. 2022; Degl’Innocenti et al. 2018). Collectively, these data confirm that dysregulation of the oxidative stress response, known to lead to severe cellular damage (Raimondi et al. 2020), plays a critical role in CCHS pathogenesis.

A more comprehensive biochemical characterisation of the oxidative imbalance is therefore required, including the quantitative assessment of specific reactive oxygen and nitrogen species as well as the evaluation of mitochondrial stress markers. Such analyses would provide deeper insight into the mechanisms by which *PHOX2B* mutations disrupt redox homeostasis and mitochondrial function.

### Cell cycle and proliferation validation

Although only small differences were detected between WT and + 13Ala, we found in all three transcriptomic datasets (sets 1–3) that PHOX2B overexpression demonstrated an effect on the cell cycle. To further investigate this observation, we performed a proliferation assay on the neuroblastoma cell line SK-N-BE transfected with an expression construct encoding PHOX2B with a GFP protein fused to the C-terminus. 

**Fig. 4:**
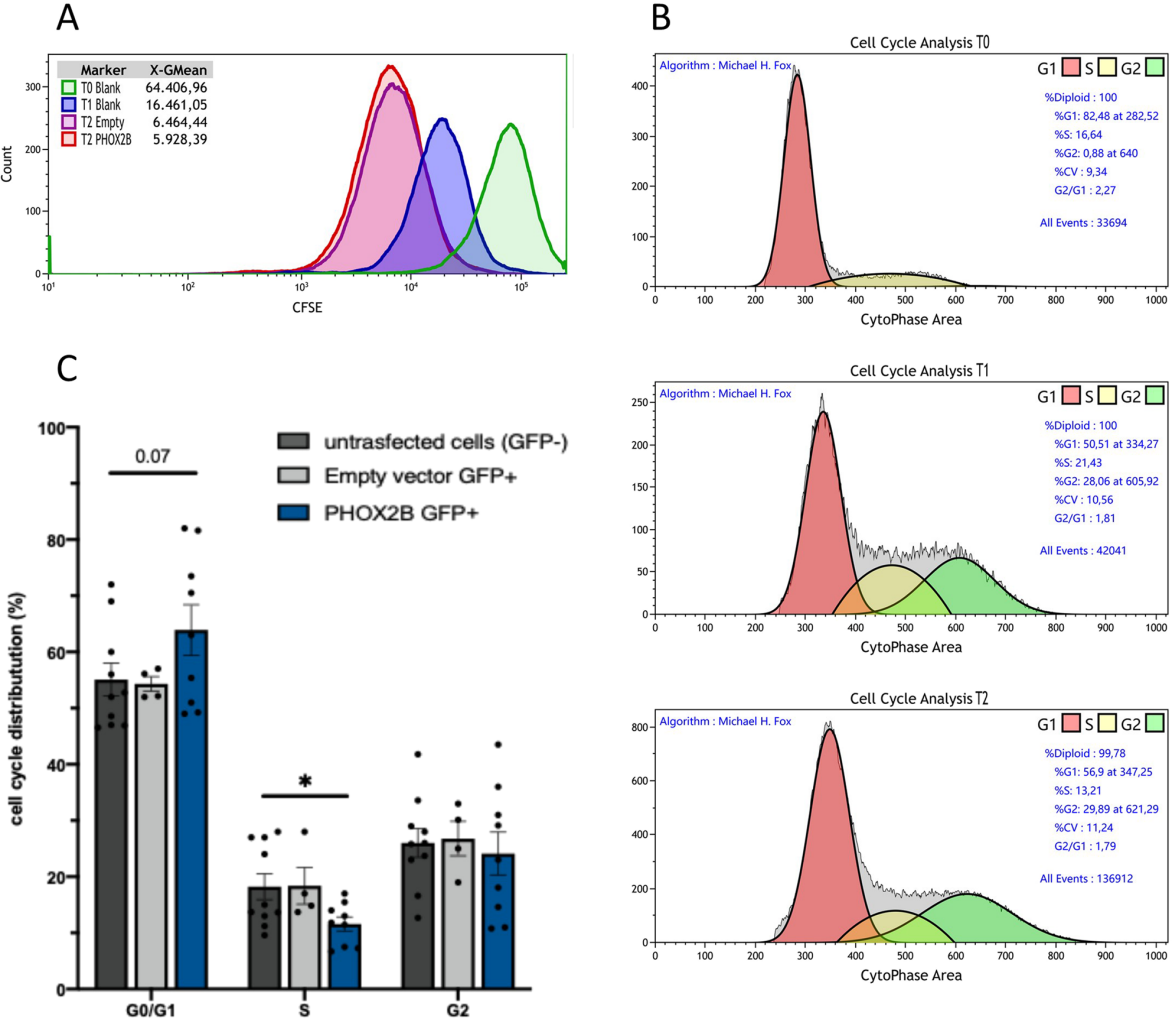
Proliferation and cell cycle evaluation after transfecting with PHOX2B. **A** proliferation assay was performed using CellTrace Far Red on synchronized cells in the G0/G1 phase. CellTrace peaks representing dye dilution during cell replication across across different time points and conditions: starved cells at the initial time point (T0shown in green), cells before transfection (T1in blue), and 24 h post-transfection with empty vector (in purple) or PHOX2B contruct(T2 in red), using a BD LSRFortessa X-20 flow cytometer. **B** Cell cycle analysis was conducted using Cytophase-Violet molecule, which intercalates with DNA. Samples were analyzed at the already presented different time points using the same instrument for the analysis and detection. Representative histograms depict the changes in cell cycle distribution of untransfected cells over time (T0, T1, and T2) based on the cellular DNA content. **C** Histogram represents the mean ± SEM of at least four independent experiments for untransfected cells (GFP-, *n* = 10), transfection control cells (empty vector GFP+, *n* = 4) and PHOX2B expressing cells (GFP+, *n* = 9) at T2. Statistical analysis was performed by two-way ANOVA with Tukey’s post hoc multiple comparisons test (*p < 0.05)

To assess potential differences in proliferation rates between PHOX2B-expressing and control cells (Fig. 4A), cell proliferation was measured using CellTrace, a derivative of the CFSE (carboxyfluorescein succinimidyl ester) fluorescent dye that covalently binds intracellular proteins and provides a long-term fluorescent signal. The fluorescence intensity histograms, with each peak corresponding to a cell generation, illustrate the progressive dilution of CellTrace as cells divide.

Cells were analysed after overnight starvation (T0), at the time of transfection (T1), and 24 h post-transfection (T2). The peaks shown in Fig. 4A represent cells under different experimental conditions (time points and transfection status). Specifically, the T0 and T1 conditions were compared with the T2 transfected samples, corresponding to cells that express the proteins encoded by the transfected constructs (empty vector or PHOX2B).

Twenty-four hours post-transfection (T2), both PHOX2B-expressing cells and cells transfected with an empty vector (GFP+) showed comparable and homogeneous proliferation rates. Furthermore, the CellTrace profile of the blank T2 sample - cells subjected to the same starvation and collection procedure but not transfected - was comparable to that of the transfected samples (data not shown). This indicates that neither the transfection procedure nor PHOX2B expression affects cell proliferation.

Although no significant differences in overall cell proliferation were observed, this could not rule out potential changes in the distribution of cells across cell cycle phases. We therefore performed a more detailed analysis of cell cycle progression. To this end, twenty-four hours post-transfection cells were stained with Cytophase that, being able to intercalate the major groove of the DNA double helix, allowed quantifying the distribution of the cells across the different cell cycle phases using flow cytometry.

The cell cycle histograms in Fig. 4B marked the cell cycle phases (G0/G1, S and G2) distribution based on the cellular DNA content. To highlight even small differences in cell cycle progression between different conditions, cells were first synchronized in G0/G1, as reported at T0 in Fig. 4B. T1 represented the point at which cells were transfected, i.e. when cells started to have a normal cell cycle increasing in S and G2 phases, and T2 was measured 24 h post transfection.

Figures 4C shows the cell cycle distribution at T2 of at least four different experiments comparing untransfected cells (GFP-), acting as negative control/blank, cells expressing the empty vector (GFP+) acting as baseline control for transfection, and PHOX2B (GFP+) transfected cells.

**Fig. 5:**
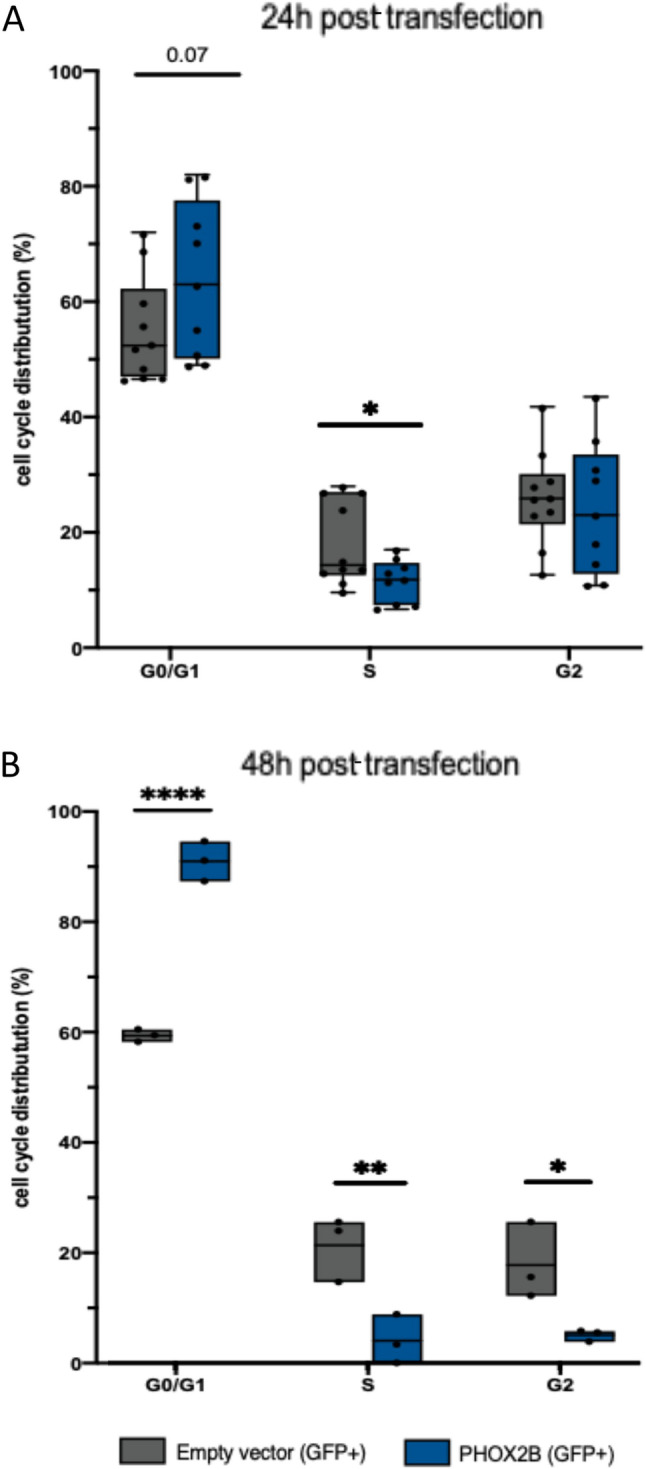
Cell cycle phases distribution at 24h and 48h post transfection. Cell cycle analysis was performed in cells untransfected (in grey) orexpressing PHOX2B contruct (in blue) at both 24hrs (panel **A**) and 48 hrs (panel **B**) posttransfection. After transfection, cells were stained withCytophase and analysed using BD LSRFortessa X-20 flow cytometer. The Histogram shows the mean ± SEM of three independent experiments.Statistical analysis was performed by two-way ANOVA with Sidak's post hoc multiple comparisons test (*p  < 0.05, **p  < 0.01, ***p  < 0.001, and ****p  < 0.0001)

Cells transfected with the empty-GFP+ vector had a similar cell cycle distribution compared to the untransfected cells, whereas the cell cycle phases were differently distributed in the PHOX2B condition. Consistently, the percentage of cells that have duplicated their DNA and are preparing for mitosis is different in cells with PHOX2B expression compared to the cells transfected with GFP control (empty vector). While non-significant, there was a trend toward increased G0/G1 cells in PHOX2B compared to other conditions. Instead, a statistically significant decrease in the percentage of S cells was revealed (*p* = 0.04), consistent with the RNA-seq results, suggesting a reduction in DNA synthesis and cell cycle delay. Although the effects observed after 24 h were modest, they likely reflect initial changes in cell cycle dynamics, given the doubling of SK-N-BE cells is considered to occur between 27 and 32 h. Therefore, to better capture alterations in cell cycle progression, we extended our analysis to a later time point, namely 48 h post-transfection. As shown in Fig. 5, which compares cell cycle effects at 24 (Fig. 5A) and 48 h (Fig. 5B) post-transfection, PHOX2B-expressing cells showed significant accumulation in G0/G1 (~ 90%) at 48 h compared to 24 h, indicating robust cell cycle arrest during cell progression. The result was highly significant when compared to cells transfected with an empty vector, which served as a baseline transfection control (****p < 0.0001). As a consequence of cell cycle arrest, after 48 h, PHOX2B-positive cells showed lower levels of cells in S and G2 phase compared to the GFP+ control (**p = 0.0014, *p = 0.013). The same trend was already evident, although less pronounced, 24 h after transfection, when cells expressing PHOX2B showed a decrease compared to control cells, although only in the S phase. However, after 48 h even PHOX2B-positive cells may have reached a high confluence, likely introducing confounding effects related to contact inhibition.

These results reflect the idea of *PHOX2B* as a key gene involved in cell cycle exit (Dubreuil et al. 2000). Cells expressing the PHOX2B construct seems to remain more in a quiescent state and a reduced percentage of cells are able to duplicate their DNA in order to start the mitotic process. The similarity between untransfected cells and empty vector (GFP+) confirm that the transfection process it is not the cause of a cell cycle impairment but it is *PHOX2B* that plays a role in cell cycle progression.

**Fig. 6:**
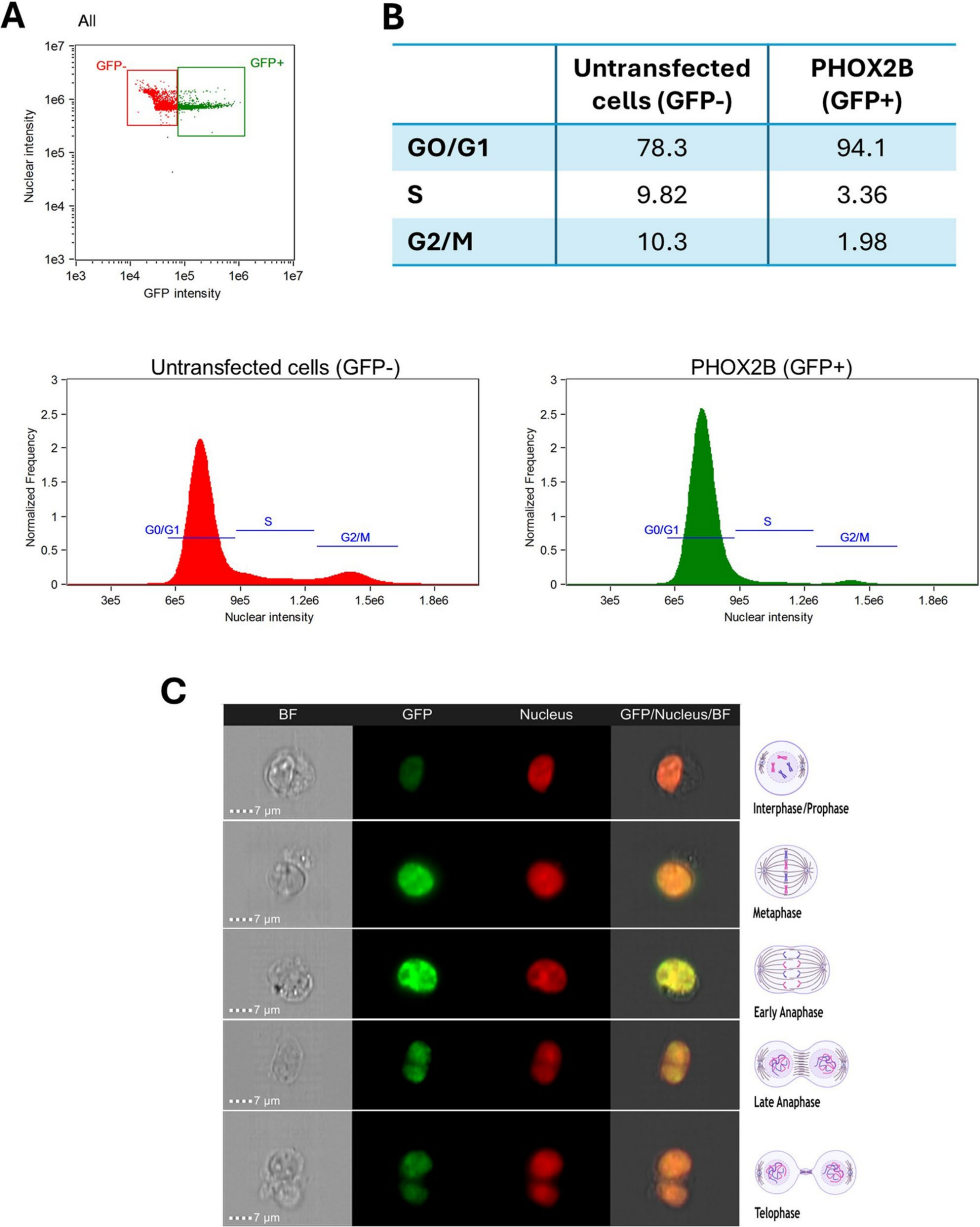
Cell cycle analysis using ImageStream instrument. Twenty-four hours post transfection, cells expressing the PHOX2B-GFP protein (GFP+) and untransfected cells (GFP-) were stained with Cytophase intercalating-DNA dye. **A** Cell-cycle profiles of GFP + and GFP- populations. **B** Quantitative distribution across G0/G1, S and G2/M phases. **C** Representative high-resolution images of cells at different stages of cell cycle. PHOX2B-GFP is shown in green and the Cytophase stained- DNA in red. Cells were classified in interphase/prophase, metaphase, early and late anaphase and telophase based on nuclear size and morphology

To further investigate the impact of PHOX2B expression on cell-cycle dynamics, we analysed PHOX2B-transfected (GFP+) and untransfected (GFP-) cells at 24 h post-transfection using the ImageStream system (Fig. 6A). This approach combines quantitative flow-cytometry measurements with high-resolution single-cell imaging. As shown in Fig. 6B, PHOX2B-expressing cells showed a clear redistribution across cell-cycle phases compared to GFP- cells. Consistent with our cytometric analysis, PHOX2B (GFP+) cells accumulated predominantly in the G0/G1 phase, with a reduction in both the S and G2/M phases, indicating a shift towards a more quiescent or controlled proliferative state, suggesting that PHOX2B expression slows or limits cell cycle progression.

Finally, Fig. 6C shows representative images of PHOX2B-expressing cells at different stages of the cell cycle, with a focus on the G2/M population captured using the ImageStream instrument. Images are shown to illustrate the ability to visually discriminate mitotic phases and to qualitatively observe PHOX2B localisation dynamics. Cells were categorised based on characteristic nuclear morphology corresponding to distinct mitotic phases. This approach could be exploited to directly assess how PHOX2B mutations affect mitotic dynamics and chromosomal organization, in line with recent reports describing PHOX2B mitotic chromatin association and its alteration in CCHS linked variants (Sato et al. 2025).

In conclusion, the cell-cycle pathway was consistently dysregulated across all datasets, as demonstrated by functional assays. Although proliferation rates did not markedly differ between groups, a higher proportion of PHOX2B-expressing cells remained in G0/G1, suggesting a role for PHOX2B in promoting quiescence or slowing cell-cycle progression. This phenotype was confirmed using two independent technologies capable of quantifying DNA intercalation through distinct approaches. PHOX2B is known to specify several classes of visceral neurons at the progenitor stage in the central and peripheral nervous systems, and also to maintain noradrenergic differentiation during embryogenesis in sympathetic ganglia (Coppola et al. 2010). Consistently, in our in vitro experiments PHOX2B has not appeared to have a role in proliferation but rather in cell cycle phases, by favouring entry into G0 and promoting cell cycle exit.

The major limitation of this study lies in the cellular model used. Since human primary cells expressing PHOX2B are not accessible, our analyses relied on transient transfection of PHOX2B constructs in neuroblastoma cells. Overexpression may introduce artefacts and may induce non-physiological stress responses. However, the reproducibility across three biological replicates and the convergence between three independent transcriptomic datasets support the robustness of our findings. Additionally, the alignment between our in vitro ROS data and the oxidative-stress signature observed in CCHS patients strengthens the relevance of our model.

Furthermore, while the separate expression of the polyAla-expanded PHOX2B protein and the WT protein, as we did in the present work, provides a valid basis for comparing their different effects, their interaction, which in vivo represents a likely key element underlying the disease, was not taken into account here. Finally, our study, focused on the pathogenic + 13Ala variant of the PHOX2B gene, cannot clarify whether the same mechanisms highlighted here also play a role in other variants associated with CCHS, such as shorter PARMs and non-PARMs.

Future studies, taking into account the interaction between expanded and WT proteins and different CCHS associated PHOX2B variants, employing more physiologically relevant systems - such as autonomous neurons derived from induced pluripotent stem cell or dental pulp stem cell, CRISPR-engineered knock-in models, or patient-derived organoids - will be essential to dissect the cell-type-specific consequences of PHOX2B mutations and to validate the mechanisms identified here (Cuadros Gamboa et al. 2022; Falik et al. 2020; Lui et al. 2023; Victor et al., 2023).

## Conclusions

This study sheds further light on the mechanisms underlying PHOX2B-mediated CCHS. In line with previous reports, the severe PHOX2B+13Ala mutation causes protein misfolding and cytoplasmic aggregation, resulting in impaired transcriptional activity and downstream gene dysregulation. Additionally, our findings reveal oxidative stress, mitochondrial dysfunction and altered cell cycle regulation as additional components of PHOX2B-mediated pathogenesis. These pathways were consistently dysregulated across multiple independent transcriptomic datasets, highlighting their biological relevance. Overall, our findings broaden our understanding of the molecular basis of PHOX2B-related CCHS and inform future studies targeting downstream pathogenic mechanisms.

## Supplementary Information

Supplementary Material 1. Supplementary Methods (additional file.docx).

## Data Availability

All data needed to evaluate the conclusions in the paper are present in the paper and/or the Supplementary Materials. The Gene Expression Omnibus (GEO) accession number for RNA-seq data is GSE250244.

## Abbreviations

CCHS: Congenital Central Hypoventilation Syndrome
PARMs: Polyalanine Repeat Mutations
17-AAG: 17-AllylAminoGeldanamycin
HDAC: Histone Deacetylase
GSEA: Gene Set Enrichment Analysis
WT: Wild-Type
DMSO: Dimethyl sulfoxide
GEP: Gene Expression Profile
UPR: Unfolded Protein Response
ETC: Electron Transport Chain
ROS: Reactive Oxygen Species
GFP: Green Fluorescent Protein
