# Supplementary material for: PHOX2B defects alter protein folding, cell-cycle, and mitochondrial pathways in an in vitro model of CCHS
Source: Mol Med. 2026 May 18;32:88. doi: 10.1186/s10020-026-01498-1 (PMC13245080; doi:10.1186/s10020-026-01498-1)
Supplement: Supplementary file 1 — Supplementary Material 1. Supplementary Methods (additional file.docx). [file 10020_2026_1498_MOESM1_ESM.pdf]

Supplementary Methods

Chiara Africano et al., **"PHOX2B defects alter protein folding, cell-cycle, and mitochondrial pathways in an in vitro model of CCHS"**

1. Cell culture

HeLa cells were selected for their well-characterized response to oxidative stress and therefore the ease with which subtle changes in ROS production can be detected. On the other hand, SK-N-BE cells are derived from human neuroblastoma and represent a relevant model for studying the transcriptome and cell cycle in a neuronal context, as expected for a CCHS model. SK-N-BE cells were maintained in RPMI medium, HeLa cells were grown in minimal essential medium (MEM, Euroclone, Italy). Each medium was supplemented with 10% fetal bovine serum (Gibco, New Zealand), 1% L-glutamine, 100 U/mL penicillin, and 100 ng/mL streptomycin (Euroclone). Cells were incubated at 37°C in 5% CO2.

2. Transfections and 17-AAG treatment

Human neuroblastoma SK-N-BE cells were selected to carry out our PHOX2B transfection experiments and subsequent gene expression analysis as expressing low levels PHOX2B ((Bachetti et al. 2010). To this end, for each condition, 250.000 cells were plated in 35mm diameter dishes in triplicate. Three sets of the following transfections and RNA extractions were performed to allow triplicates and reliable statistical analysis of the gene expression levels and enrichment profiles (from now on called "Set #1"). In particular, three micrograms of plasmids pcDNA3.1Myc-*PHOX2B* WT or pcDNA3.1Myc-*PHOX2B* +13Ala were transfected by using Fugene HD (Promega), as already reported (Africano et al, 2024). Twenty-four hours later, cells were treated with 17-AAG 1.0 mM stock in DMSO, diluted into fresh medium to 300nM final concentration, or left untreated. Forty-eight hours after transfections RNAs were extracted from the four cell cultures for the transcriptome analysis and protein lysates were prepared for the validation assays.
Two independent experiments were carried out using the protocol described above for SK-N-BE cells, which were either untransfected or transfected with the empty control vector, the WT version of the *PHOX2B* gene (pcDNA3.1Myc-*PHOX2B* WT), or the +13Ala version of the *PHOX2B* gene (pcDNA3.1Myc-*PHOX2B* +13Ala). These two datasets (from now on called "Sets #2" and "Set #3") were used to confirm the results obtained in Set #1.

3. **RNA extraction and transcriptome analysis**

Total RNA was extracted and purified with RNeasy Plus mini kit (Qiagen) and RNA samples thus obtained were quantified by NanoDrop (Thermo Scientific, Rockford, USA). The quality and amount of RNA samples were verified by the Agilent Technologies 2100 Bioanalyzer using RNA 6000 LabChip ® kit (Agilent#5067-1511). mRNA was isolated from 200 ng of total RNA using poly-T oligo-attached magnetic beads. Purified samples were processed using Illumina TruSeq Stranded mRNA library preparation kit. Indexed individual libraries were pooled to obtain equimolar concentrations for each sample, and then loaded on a single-end flow cell of HiSeq 2500 instrument (Illumina) to generate 30 million of 75bp single-end reads per sample.

The quality of the RNA-Seq experiments was evaluated using RSeQC (Wang et al. 2012) tools. Transcript abundance was estimated with Kallisto using Ensembl transcripts GRCh38 (Bray et al. 2016) including the count of PHOX2B reads and differentially expressed (DE) genes were identified using the DeSeq2R package (Love et al. 2014) R package. We controlled for batch effect while testing for associations due to condition by adding the experimental batch in the design formula (~batch + condition). Differentially expressed (DE) genes were determined considering False Discovery Rate (FDR) corrected p-values < 0.05.

4. **Gene set enrichment analysis (GSEA)**

Gene set enrichment analysis (GSEA) (Subramanian et al. 2005) is widely applied to determine whether a predefined gene set shows statistically significant difference between two biological states. We applied GSEA in order to identify gene sets and pathways which were significantly perturbed across conditions. Collections of gene sets were downloaded from MSigDB v7.4 [1]. Pathways were selected if they showed at least one significant comparison between conditions using a FDR < 0.05 as the significant threshold. Details on these procedures have already been reported (Africano et al. 2024).

5. **ROS evaluation**

$2x10^5$ HeLa cells were plated in 35 mm Petri dish and transfections were performed after 24 hours using FuGeneHD (Promega) in a ratio 3:1 with 1,5 µg of expression vectors

pcDNA3.1Myc-PHOX2B WT and pcDNA3.1Myc-PHOX2B +13Ala fused with the Myc sequence.

Forty-eight hours post-transfection, hypoxia and oxidative stress (ROS) levels were measured in protein lysates by flow cytometry (FACSCanto II- BD Biosciences) using ROS-ID® Hypoxia/Oxidative Stress Detection Kit (Enzo Life Sciences, Farmingdale, NY, USA) following the manufacturer's instructions. Analysis was performed using the Kaluza 2.1 software (Beckman Coulter).

6. **Cell cycle and proliferation analysis**

SK-N-BE were induced to starvation overnight with RPMI 1% penicillin/streptomycin and L-glutamine without serum. The day after, cells were detached and incubated 20 mins with CellTrace Far Red (1:1000, Thermo Fisher) in 1 ml of warm PBS and then $1.5x10^5$ SK-N-BE cells were plated in 35 mm Petri dish. Transfection was performed 24 h later using FuGeneHD (Promega) in a ratio 3:1 with 1.5 µg of expression vectors pcDNA3.1CT-GFP, pcDNA3.1CT-GFP *PHOX2B WT*, pcDNA3.1CT-GFP *PHOX2B+13Ala* fused with the GFP sequence. The empty vector, expressing only the GFP protein without PHOX2B, was transfected as a cell cycle control.

For cell cycle analysis three different points were analysed corresponding to. cells after the overnight starvation (T0), at the time of transfection (T1) and twenty-four h after the transfection (T2). Cell cycle was analysed adding 1:700 of Cytophase-Violet (Biologend) per condition followed by one hour incubation at 37°C. Furthermore, an additional time point at 48 h post-transfection was also included using the same protocol. Treated cells were then analyzed for cell cycle and proliferation analysis by flow-cytometry (BD LSRFortessa X-20) and ImageStream XII.

7. **Statistical analysis**

Statistical analysis was performed using GraphPad Prism. A two-way analysis of variance (two-way ANOVA) was applied to access differences in ROS production under hypoxic conditions and in cell cycle distribution. Multiple comparisons were performed using Sidak's or Tukey's post hoc tests, as appropriate. Differences were considered statistically significant at $p < 0.05$ (∗p < 0.05, ∗∗p < 0.01, ∗∗∗p < 0.001, and ∗∗∗∗p < 0.0001).

REFERENCES

Africano C, Bachetti T, Uva P, Pitollat G, Del Zotto G, Giacopelli F, et al. Identification of a histone deacetylase inhibitor as a therapeutic candidate for congenital central hypoventilation syndrome. Mol Ther Nucleic Acids 2024;35. https://doi.org/10.1016/J.OMTN.2024.102319.

Bachetti T, Di Paolo D, Di Lascio S, Mirisola V, Brignole C, Bellotti M, et al. PHOX2B-mediated regulation of ALK expression: in vitro identification of a functional relationship between two genes involved in neuroblastoma. PLoS One 2010;5. https://doi.org/10.1371/JOURNAL.PONE.0013108.

Bray NL, Pimentel H, Melsted P, Pachter L. Near-optimal probabilistic RNA-seq quantification. Nat Biotechnol 2016;34:525–7. https://doi.org/10.1038/NBT.3519.

Love MI, Huber W, Anders S. Moderated estimation of fold change and dispersion for RNA-seq data with DESeq2. Genome Biol 2014;15. https://doi.org/10.1186/S13059-014-0550-8.

Subramanian A, Tamayo P, Mootha VK, Mukherjee S, Ebert BL, Gillette MA, et al. Gene set enrichment analysis: a knowledge-based approach for interpreting genome-wide expression profiles. Proc Natl Acad Sci U S A 2005;102:15545–50. https://doi.org/10.1073/PNAS.0506580102.

Wang L, Wang S, Li W. RSeQC: quality control of RNA-seq experiments. Bioinformatics 2012;28:2184–5. https://doi.org/10.1093/BIOINFORMATICS/BTS356.

1. Gene Set Enrichment Analysis (GSEA). https://www.gsea-msigdb.org/gsea/index.jsp. Accessed 19 February 2026.
